# CD20-positive NK/T-cell lymphoma with indolent clinical course: report of case and review of literature

**DOI:** 10.1186/1746-1596-7-133

**Published:** 2012-10-02

**Authors:** Qing-ping Jiang, Shao-yan Liu, Yue-xin Yang, Xue-xian Tan, Juan Peng, Zhong-tang Xiong, Zhi Li

**Affiliations:** 1 Department of Pathology, the Third Affiliated Hospital of Guangzhou Medical University, 63, Duobao Road, Guangzhou, 510150, China; 2 Department of Pathology, The First Affiliated Hospital, Sun Yat-sen University, 58, Zhongshan Road II, Guangzhou, 510080, China

**Keywords:** NK/T cell lymphoma, Nasal type, CD20 expression, Differential diagnosis, Prognosis

## Abstract

**Abstract:**

CD20-positive T-cell lymphoma is extremely rare and only two cases of CD20-positive NK/T-cell lymphoma with aggressive clinical courses have been described in the literature. We present a case of unusual NK/T-cell lymphoma with CD20 expression in nasal cavity occurring in an elder female patient. The patient had presented with left nasal cavity nodule for 10 years. CT scan revealed a mass was located at the left anterior nasal cavity and was observed to extend into the ethmoid sinus. There was no regional lymph node involvement. Biopsy was performed and microscopical inspection revealed the lesion was composed of small- to middle-size atypical lymphoid cell, histiocytes, eosinophils, and neutrophils. The lymphoid cells were strongly immunoreactive to CD3, CD20, CD56, TIA-1 and granzyme-B. The Epstein-Barr virus genomes were also found in tumor cells by in situ hybridization. By genetic analysis, however, no clonal rearrangement of the T cell receptor-γ genes (TCRG), or the immunoglobulin heavy chain (IgH) gene was found. A diagnosis of CD20-positive extranodal NK/T-cell lymphoma, nasal type was made. The patient refused chemotherapy, and had been only on regular follow-up for 6 months. There was no sign of enlargement of tumor and extra-nasal dissemination by whole body positron emission tomography/computed tomography (PET/CT) study. The accurate diagnosis of NK/T-cell lymphoma with CD20 expression is important, but the indolent behavior of the present case is more unusual. A long-term follow-up is suggested to be performed to inspect the progression for this tumor.

**Virtual slides:**

The virtual slides for this article can be found here: http://www.diagnosticpathology.diagnomx.eu/vs/1320848277788495

## Background

Immunophenotyping is an integral part of lymphoma diagnosis and immunohistochemistry is one of most important methods to be used for classification of lymphomas [1]. As a diagnostic marker, CD3 and CD20 are most widely used for T- and B-cell lineage, respectively. These markers have long been thought to be specific and used to help differentiate T-cell and B-cell neoplasms. In the overwhelming majority of cases, B- or T-cell lymphomas do not express opposite markers, but co-expression of T- and B-cell markers can also be found in some subtypes of lymphomas, such as T lymphoblastic leukaemia/lymphoma (CD79a positivity has been observed in approximately 10% of cases) [2], small lymphocytic lymphoma/chronic lymphocytic leukaemia and mantle cell lymphoma (CD5-positive in tumor cells represent as typical immunohistochemical characteristics) [3]. However, the expression of CD20 in a T-cell lymphoma is extremely rare. To our best knowledge, so far 39 cases of CD20-positive T-cell lymphoma [4-25] and only 2 cases of CD20-positive NK/T-cell lymphoma [26,27] have been previously described in the literature. Since the presence of CD20 is generally considered specific for B-lineage on both benign and neoplastic lymphocytes, the accurate diagnosis of a T-cell lymphoma with CD20 expression is quite difficult and challenging. Herein, we report an additive CD20-positive NK/T-cell lymphoma occurring in the nasal cavity of an elder female patient. In contrast to most of previously reported cases with aggressive behavior, our case presents an indolent clinical course with 10 years of duration. The clinical and histological features of this tumor, as well as differential diagnosis are discussed.

### Case presentation

#### Clinical presentation and management

**Figure 1:**
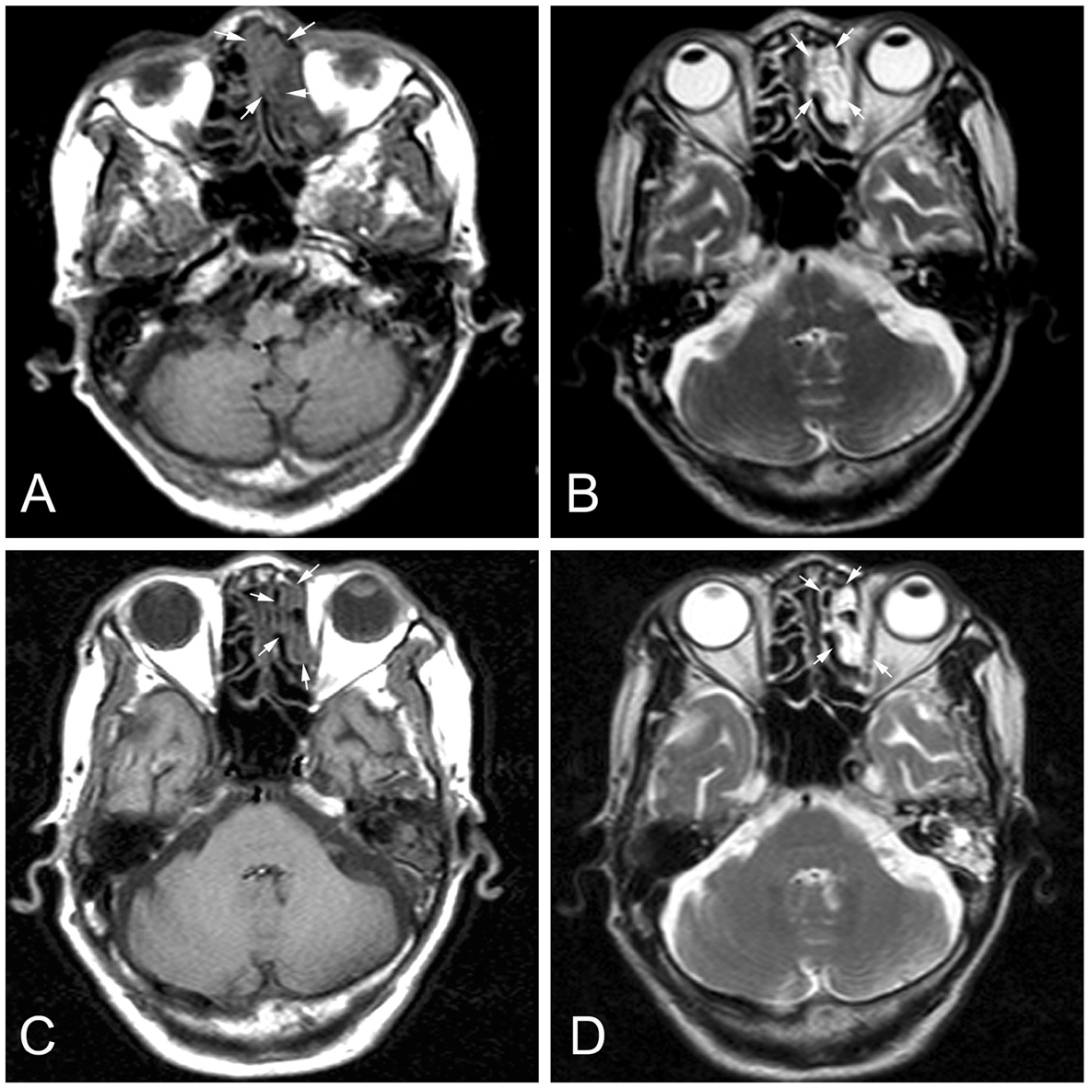
**Radiological findings of the intranasal mass (A) Axial CT scan (T1) revealed that an irregular mass presented in the left nasal cavity displacing the nasal septum (white arrow).** (**B**) CT scan (T2) showed that the mass also extended into left ethmoid sinus, but did not erode the bony margins of the medial wall of the left orbit (white arrow). After 6-month period of follow-up, axial CT scan T1 (**C**) and T2 (**D**) showed that location and size of the mass did not change remarkably (white arrow).

A 78-year-old female patient had presented with complaints of mild headache, left nasal obstruction and rhinorrhea for 10 years. The patient had been referred to a local clinic and treated with antibiotics for “rhinitis and nasal polyp” for several times, but the symptoms were not improved. At that time, a small “polyp-like” nodule was found in the left anterior nasal cavity, but neither radiological examination nor biopsy was performed. During that period, the patient developed fatigue and had gradually weakened smell acuity. Three months before admission to our hospital, she was suffering from bloody rhinorrhea and severe headache. As a result, the patient was referred to our hospital for examination and treatment. Physical examination showed that the left anterior nasal cavity was obstructed by a large reddish mass with amount of purulent exudation. The mass filled the left nasal cavity and compressed the nasal septum to the opposite side. There was no defect in visual field test and vision acuity in her eyes. The laboratory results, including blood count, differential, liver and renal function, were within the normal range. There was no weight loss and no palpable lymphadenopathy or organomegaly. Computerized tomographic (CT) scans revealed an irregular, homogeneous mass in the left nasal cavity measuring 1.5 cm×1.0 cm. The mass occupied the whole left nasal cavity and was observed to extend into the ethmoid sinus (Figure 1A). The biopsy of the left nasal mass was performed. After diagnosis, the patient refused the chemotherapy and was only on regular follow-up. A endoscopic examination at the end of 6 months follow-up period showed that a mass was at the site of original tumor location. The CT scan revealed that the size of nasal mass did not change remarkably and no enlarged lymph node was observed (Figure 1B). Since there was a possibility of dissemination to another anatomical location, the patient was referred to a whole body positron emission tomography/computed tomography (PET/CT) study to search for a potential secondary tumor, but no abnormality was found. The patient was until now on regular follow-up.

## Material and methods

The biopsy tissue was routinely fixed in 10% neutral buffered formalin and embedded in paraffin. Four micrometer-thick sections were stained with hematoxylin and eosin. Immunohistochemical analyses were performed using the ChemMate Envision/HRP Kit (Dako, Glostrup, Denmark). The antibodies used in this study were CD2, CD3, CD4, CD8, CD56, CD30, TIA-1, granzyme-B, CD20, CD138, CD68, CD79a, TdT, MPO, and ki-67. The antibodies were obtained from Dako Cytomation (Carpinteria, CA) and Santa Cruz Biotechnology (Santa Cruz, CA). Slides were dewaxed and rehydrated routinely and then were treated with 10 mmol citrate buffer (pH 6.0) in a microwave for antigen retrieval. After incubation with diluted primary antibodies, slides were treated with the ChemMate Envision/HRP Kit for 30 minutes at room temperature followed by development with diaminobenzidine (DAB) for visualization.

For detection of Epstein-Barr virus (EBV) infection in the tissues, in situ hybridization for EBERs (EBV-encoded RNAs) was performed on the biopsy. The EBERs detection kit was purchased from Dako (Glostrup, Denmark). The detection process was conducted according to the manufacturer’s instructions.

For cytogenetic analysis, the paraffin tissue DNA was prepared with a tissue DNA extraction and purification kit (DneasyTM Tissue Kit, Qiagene, CA). T-cell receptor and immunoglobulin gene rearrangement studies were performed. Two sets of primers (tube A, 145–255 bp; tube B, 80-220bp) were used to amplify the rearranged T-cell receptor (TCR)-γ gene. A T cell lymphoma case with a known monoclonal rearrangement was used as the positive control, a non-lymphoid and hematopoietic tumor was used as the negative control, and a reaction without template DNA was simultaneously run as the blank control. β-actin was amplified as an internal control. FRIII-J segment were conducted for IgH gene rearrangements. RAJI cells were used as positive controls and a previous negative sample was used as a negative control. The detection process was conducted by the methods previously described.

### Pathological findings

**Figure 2:**
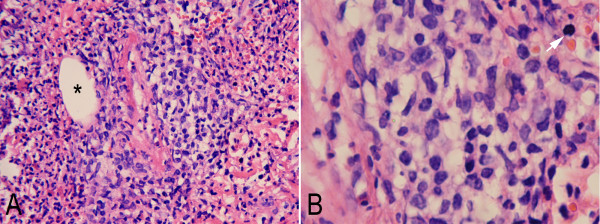
**Photomicrographs of the intranasal mass. **(**A**) Low-power view showed the mass was mainly infiltrated by small- to middle-size atypical lymphoid cell with admixed inflammatory cells. Perivascular infiltrating and local angioinvasion (*) were observed in the lesion. (**B**) At higher magnification tumor cells showed irregular nuclear borders and had variable amounts of cytoplasm. Mitotic figures were scattered throughout the lesion (white arrow) (A, HE staining with original magnification ×100; B, HE staining with original magnification ×400).

**Figure 3:**
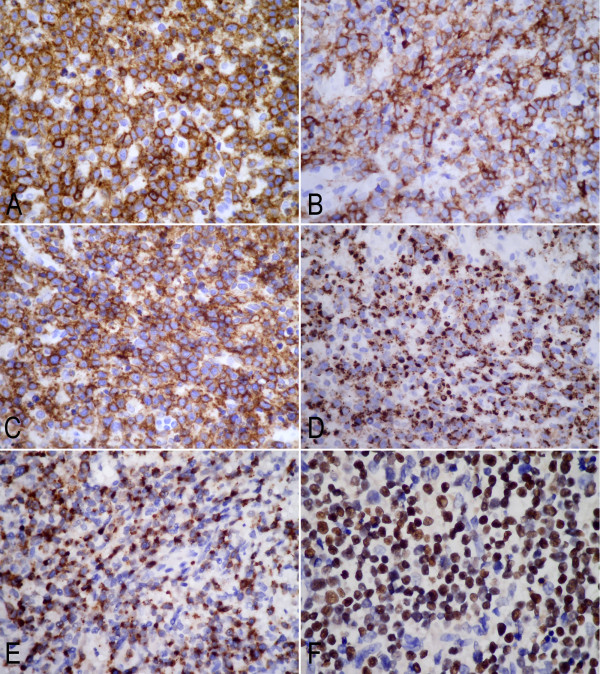
**Immunohistochemical analysis of the intranasal mass showed tumor cells were diffusely positive for CD3 (A), CD20 (B), CD56 (C), granzyme B (D) and TIA-1(E). **(**F**) Tumor cells were also positive for Epstein-Barr virus small-encoded RNA (EBERs) by in situ hybridization (**A**-**E**, immunohistochemical staining with original magnification ×400; **F**, in situ hybridization for EBERs with original magnification ×400).

Under a microscope, the mass showed extensive necrosis and inflammatory exudation. The lesion was mainly infiltrated by small- to middle-size atypical lymphoid cell, histiocytes and eosinophils. Tumor cells showed irregular nuclear borders and had variable amounts of cytoplasm. Mitotic figures were scattered throughout the lesion. Perivascular infiltrating and local angioinvasion were noted in the lesion (Figure 2). Immunohistochemical staining showed that tumor cells were strongly positive for T cell markers, CD2, and CD3, focally positive for CD4. Co-expression of the B cell marker CD20 was noted in most tumor cells, but other B cell markers, including CD79a, PAX5, and CD138, were negative. The tumor cells were also positive for the natural killer (NK) cell marker CD56 and the cytotoxic marker, CD8, TIA-1 and granzyme B strongly and diffusely, but negative for CD30, CD68, TdT, and MPO. Ki-67 index was approximately 60%. The EBERs probe, distinct positive signals were demonstrated diffusely in the nuclei of tumor cells in the lesion by in situ hybridization (Figure 3). However, there was no clonal rearrangement of the T cell receptor gamma (TCRG) or the immunoglobulin heavy chain (IGH) genes found in the lesion by cytogenetic analysis. The clinical and histopathological finding of this case was in accord with the extranodal NK/T cell lymphoma, nasal type.

## Conclusions

The expression of CD20 in tumor cells of T-cell lymphomas is quite rare but well-recognized phenomenon. We have reviewed 39 cases of CD20-positive T-cell lymphoma reported in the English literature [4-25]. It reveals that the majority of cases are classified as peripheral T-cell lymphoma, unspecified (25/39 cases, 64.1%), followed by T-cell lymphocytic leukemia (7/39, 17.9%), mycosis fungoides (3/39, 7.7%), anaplastic large cell lymphoma (2/39, 5.1%), T-cell lymphoma with features of angioimmunoblastic T-cell lymphoma (1/39, 2.6%) and enteropathy-type T-cell lymphoma (1/39, 2.6%). Most patients are elderly males (range from 3 to 84 years at diagnosis) and many cases behave aggressively. The tumor cells are positive for one or more pan-T-cell antigens (CD2, CD3, CD5, or CD7) and CD20 with monoclonal rearrangements of TCR γ or β without rearrangement of the IgH gene. CD79a, another B cell marker, can also be expressed as well as CD20 in some cases [12,21]. In the present case, we found that the tumor cells exhibited co-expression of CD20 and markers for T-cell lineage and NK cell lineage, but were negative for TCR and IgH gene rearrangements. Since NK/T-cell neoplasms typically lack clonal TCR gene rearrangements and Vβexpression [28], our case is consistent with a typical extranodal NK/T-cell lymphoma, nasal type with CD20 expression.

We have reviewed 2 cases of CD20-positive NK/T-cell lymphoma in the literature (Table 1). Both of them are adults and from East Asia initially presenting with subcutaneous mass in unusual sites. In the present case, immunohistochemical and molecular analysis definitely concluded a typical extranodal NK/T-cell lymphoma. To our knowledge, it is the third case of CD20-positive NK/T-cell lymphoma. However, in contrast to previous cases, our case presents an indolent clinical course with long-term duration. It has revealed that NK/T-cell lymphoma may be predominantly localized or may be disseminated at initial examination with an aggressive behavior [28]. Although NK or T lineage of tumor cells has no impact on patient survival [29], dissemination to multiple organs, such as liver, spleen, skin, and/or bone marrow at presentation was the most important factor predicting poor outcomes [30]. Therefore, nasal cavity-only lesion in our case might be responsible for its indolent clinical course and favorable prognosis. Till date, to our knowledge, only 2 cases of CD20-positive T-cell lymphoma had indolent courses. Rahemtullah et al. demonstrated that a case of CD20-positive T-cell lymphoma died 66 months after the original diagnosis [21]. Xiao et al. also reported a case of CD20-positive T-cell lymphoma with 12-year history of lymphoma [24]. These findings raise a question of whether or not CD20-positive T-cell or NK/T-cell lymphomas have a somewhat smoldering stage before aggressive clinical courses, although CD20 expression in T-cell lymphomas does not appear to affect clinical behavior in most reported cases. In our study, the patient was diagnosed as NK/T-cell lymphoma after 10-year onset of initial manifestation. Moreover, the nasal cavity mass kept silence over a 6-month period without any treatment. We postulated that this patient might be still in the smoldering stage of tumor. Once the tumor progress from smoldering stage to active lymphoproliferative stage, dissemination of extra-nasal sites might be presented and the patient might gain a poor prognosis with aggressive clinical course. Of course, long-term follow-up should be performed to verify this postulation.

**Table 1:** Clinicopathological features of patients with CD20-positive NK/T-cell lymphoma described in present and previous reports

**No.**	**Authors (yr.)**	**Diagnosis**	**Age (year)/ Gender**	**Clinical Presentation**	**Immunophenotype**	**Molecular analysis**	**Treatment**	**Outcome**
1	Ando, et al. (2008) [26]	Extranodal NK/T cell ymphoma, nasal type	71/Male (Japanese)	Ulcerative mass in the right thenar prominence and a subcutaneous mass in right inguinal region	CD20+, CD2+, CD3+, CD56+, TIA-1+, granzyme B+, CD4-, CD5-, CD7-, CD8-, CD10-	EBERs (+); TCR β (−); TCR γ(−); TCR δ (−); IgH (−)	Chemotherapy with CHOP, ESHAP, and L-asparaginase	Progressive disease without remission and death in 6 months
2	Gill, et al. (2010) [27]	Disseminated NK/T cell lymphoma	25/Male, (Chinese)	Hypermetabolic lesion in right chest wall, head and neck lymph node, left adrenal gland, peritoneum, liver and the right anterior nasal cavity	CD20 focal +, CD2+, CD45RO+, CD56+, TIA-1+, CD4-, CD5-, CD8-, CD79a-, PAX5-, Oct2-, BOB.1-, CD138-	EBERs (+); TCR β (−); TCR γ(−); IgH (−)	Not reported	Not reported
3	Present case	Extranodal NK/T cell lymphoma, nasal type	78/Female (Chinese)	A solid mass in the left nasal cavity for 10 years	CD20+, CD2+, CD3+, CD56+, CD8+, TIA-1+, granzyme B+, CD79a-, CD30-, PAX5-, CD138-	EBERs (+); TCR γ(−); IgH (−)	No treatment	6-months follow-up, alive

As a specific B-cell marker, CD20 has been used to distinguish B-cell from T-cell lymphoma. It is a 35-kDa transmembrane protein expressed from early pre-B-cell development until terminal differentiation into plasma cells. Several hypotheses have been proposed to explain the nature of CD20-positive T cell lymphoma, including normal circulating CD20 positive T cells undergoing neoplastic transformation [31], a marker of normal T cell activation [32], and neoplastic T cells aberrantly acquiring CD20 positivity [12,22]. A recent study suggests that in some cases, CD20 positive may represent neoplastic transformation of an activated T-cell subset that it has variable CD20 expression, whereas in other cases CD20 may be an activation marker acquired after neoplastic transformation [21]. It is well known that the existence of CD20-positive T cells in the peripheral blood of healthy individuals [33], and two-thirds of these normal CD3+CD20+ T cells are CD8 positive and one-third is CD4 positive in the peripheral blood. Moreover, T cells and NK cells share the same ontogeny from a common progenitor cell, accounting for the frequent expression of NK cell antigens on T cells and vice versa. In the present case, the CD20-positive cells showed cellular atypia with co-expression of T-cell markers. However, CD30, another activation marker, was not detected in tumor cells. If CD20-positive in T-cell is indeed an activation marker, we consider it might be along with increased expression of CD30. Therefore, we prefer to accept the hypothesis of neoplastic transformation of a normal subset of CD20-positive T-cells rather than an activation marker acquired after neoplastic transformation.

Despite its enigmatic histogenesis, the significance of cross-lineage antigen expression in this tumor might confuse the diagnosis. Sun et al. point out that flow cytometry analysis is useful in making the distinction between B and T-cell lymphomas because CD20-positive T-cell lymphomas tend to be CD5 bright and CD20 dim, while CD5-positive B-cell lymphomas tend to be CD5 dim and CD20 bright. However, this difference of staining intensity may be difficult to appreciate under the microscope [16]. Therefore, for the histopathologists, a large immunohistochemical panel might lead to avoid misdiagnosis. The application of CD20 and CD79a as B-cell markers and CD3 and CD5 as T-cell markers is currently recommended for lymphoproliferative diseases. In addition, molecular analysis is useful for differential diagnosis, because it is very difficult to diagnose T-cell lymphomas without evidence of clonal TCR-γ or -β chain gene rearrangements. For CD20-positive NK/T-cell lymphoma, detection of EBERs by in situ hybridization is suggested to be essential for accurately diagnosing this small/medium-sized lymphoma.

In conclusion, only a few cases of CD20-positive NK/T-cell lymphoma have been reported in the literature. Our additive case is also presented for its rarity of immunophenotype and its unusual clinical manifestation. It is the first case of CD20-positive NK/T-cell lymphoma with an indolent clinical course. The diagnosis of CD20-positive T-cell or NK/T-cell lymphoma is difficult and should be made cautiously. Besides confirmation by strict histopathological and a large panel of immunohistochemical analysis, molecular analysis must be essential to accurately diagnose those neoplasms with disordered and unusual immunohistochemical features.

### Consent

Written informed consent was obtained from the patient for publication of this case report and any accompanying images. A copy of the written consent is available for review by the Editor-in-Chief of this journal.

## Abbreviations

NK: Natural killer; TCR: T-cell receptor; EBV: Epstein-Barr virus; EBERs: EBV-encoded RNAs; IGH: Immunoglobulin heavy chain.

## Competing interests

The authors declare that we have no competing interests.

## Authors' contributions

QP J and SY L made contributions to acquisition of clinical data, and analysis of the histological features by H&E staining. J P and ZT X carried out the immunoassays and molecular analysis. YX Y and XX T drafted the manuscript. ZL revised manuscript critically for important intellectual content and had given final approval of the version to be published. All authors read and approved the final manuscript.
